# Case Report: Whole exome sequencing identifies variation c.2308G>A p.E770K in *RAG1 *associated with B- T- NK+ severe combined immunodeficiency

**DOI:** 10.12688/f1000research.9473.2

**Published:** 2017-10-02

**Authors:** Geeta Madathil Govindaraj, Shamsudheen Karuthedath Vellarikkal, Rijith Jayarajan, Rowmika Ravi, Ankit Verma, Krishnan Chakkiyar, Machinari Puthenpurayil Jayakrishnan, Riyaz Arakkal, Revathi Raj, Rajeevan Kunnaruvath, Sridhar Sivasubbu, Vinod Scaria

**Affiliations:** 1 Department of Pediatrics, Institute of Maternal and Child Health, Government Medical College, Kozhikode, India; 2 Academy of Scientific and Innovative Research (AcSIR), CSIR-IGIB, Delhi, India; 3 Genomics and Molecular Medicine Unit, CSIR Institute of Genomics and Integrative Biology, Delhi, India; 4 Apollo Speciality Hospital, Chennai, India; 5 Department of Pathology, Government Medical College, Kozhikode, India; 6 GN Ramachandran Knowledge Center for Genome Informatics, CSIR Institute of Genomics and Integrative Biology, Delhi, India

**Keywords:** Severe Combined Immunodeficiency, B- T- NK+ SCID, Whole Exome Sequencing, RAG1

## Abstract

Severe combined immunodeficiency is a large clinically heterogeneous group of disorders caused by a defect in the development of humoral or cellular immune responses. At least 13 genes are known to be involved in the pathophysiology of the disease and the mutation spectrum in SCID has been well documented. Mutations of the recombination-activating genes RAG 1 and RAG 2 are associated with a range of clinical presentations including, severe combined immunodeficiency and autoimmunity. Recently, our understanding of the molecular basis of immune dysfunction in RAG deficiency has improved tremendously with newer insights into the ultrastructure of the RAG complex. In this report, we describe the application of whole exome sequencing for arriving at a molecular diagnosis in a child suffering from B- T- NK+ severe combined immunodeficiency. Apart from making the accurate molecular diagnosis, we also add a genetic variation c.2308G>A p.E770K to the compendium of variations associated with the disease.

## Introduction

Severe combined immunodeficiency (SCID) encompasses a constellation of clinically and genetically heterogeneous diseases resulting in defects of the humoral and/or cellular immune defense mechanism ^1^. The patients with SCID exhibit recurrent infections with bacteria, virus and fungi. The deficiency of recombination activating gene is associated with T ^-^B ^-^NK ^+^ SCID. Recombination of activating gene enzymes plays a significant role in recombination of V(D)J segments ^2^. The mutation in recombination activating gene 1 ( *RAG1*) is associated with absence of V(D)J recombination, which in turn produces immature lymphocytes leading to SCID ^3^. The accurate molecular diagnosis in SCID enables genetic counselling for disease ^4^. So far, arriving at a precise molecular diagnosis has been quite cumbersome, technically challenging and expensive, as over a dozen genes are known to be implicated in the genetic disease, which would require systematic targeted sequencing of each of the gene ^5, 6^. The advent of next generation sequencing, especially whole exome and sometimes whole genome sequencing has significantly enabled the rapid identification of the causative genetic variations in clinical settings ^6^.

In this report, we describe the application of whole exome sequencing for the accurate molecular diagnosis of a case of T ^-^B ^-^NK+ SCID. Our report also adds a genetic variation c.2308G>A p.E770K in Recombination activating gene 1 ( *RAG1*) to the compendium of variations associated with the disease.

## Case report

Here we report a case of a seven-month-old boy, born out of a third degree consanguineous marriage, with a history of recurrent episodes of pneumonia, acute otitis media, diarrhea and oral thrush since two months of age. The child was pale, emaciated, febrile, and had respiratory distress with lower chest retractions. He was in compensated shock. There was no clubbing, cyanosis or lymphadenopathy. There was no facial dysmorphism, and skin and hair were normal. He weighed 5.4 kg; measured 64 cm in length and head circumference was 39.5 cm; all below the 3rd centile as per WHO Child Growth Standards. Examination of the chest showed evidence of bronchopneumonia while there was no evidence of congenital heart disease or neurological deficits. There was mild hepatomegaly with a liver span of 6.5 cm.

**Figure 1.**
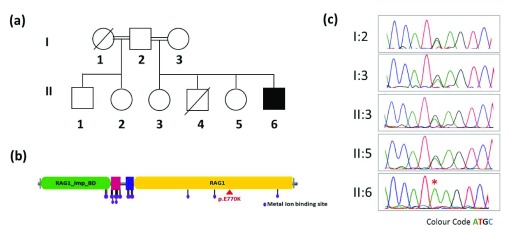
( **a**) Pedigree of the family ( **b**) domain mapping of the RAG1 p.E770K on RAG1 protein showing the variation lies on RAG1 domain highlighted with red triangle and ( **c**) capillary sequencing of the locus in the proband and family members. The homozygous variation c.2308G>A in the proband is marked with an asterisk.

The baby was normal in the perinatal and postnatal period. His birth weight was normal (3.04 kg) and was asymptomatic until 2 months of age. There was a history of admission to pediatric intensive care unit (PICU) and artificial ventilation for severe pneumonia at the age of 2 months. He was admitted for 22 days during that episode. The child had gross motor developmental delay and no adverse events following immunization. He had a male sibling who expired at seven months of age due to persistent pneumonia and two unaffected female siblings, apart from a half-brother and half-sister both of whom were asymptomatic ( Figure 1A).

On investigation, the child was found to have hypochromic microcytic anemia, lymphocytopenia, and a normal eosinophil count and platelet count. Liver and renal function tests were normal. The ionized calcium was 0.28mmol/L. Blood culture was positive for Enterobacter species. The child’s mother was HIV ELISA negative and the child had a negative Mantoux test and negative gastric acid AFB stain. His chest X-Ray’s lateral view showed absence of the thymus shadow apart from evidence of bronchopneumonia.

A close workup of the immunoglobulin profile revealed hypogammaglobulinemia-IgA 23 mg%, IgG 44 mg% and IgM 26 mg%. IgE was 1 IU/L. The absolute CD3 count was 464 cells/ul (normal range 1,460–5,440 cells/ul), absolute CD19 lymphocyte count was 12 cells/ul (normal 430–3,300 cells/ul) and absolute NK cell count was 1,328 cells/ul (normal 80–340 cells/ul). Flow cytometry suggested absent B and markedly reduced T cell populations suggestive of B- T- NK+ SCID.

The child was treated with piperacillin (80mg/kg/dose Q8H), vancomycin (15 mg/kg/dose Q6H), dopamine (10 mic/kg/min), intravenous immunoglobulin (IVIG) and other supportive measures and was put on cotrimoxazole (6 mg/kg/day OD) prophylaxis. He was treated with ganciclovir for CMV infection and for staphylococcal pneumonia.

The child was referred for a bone marrow transplant, since SCID is not compatible with life beyond infancy. The patient underwent a matched sibling donor bone marrow transplant at the age of 1 year and 3 months. The conditioning regimen used was Fludarbine 40mg/M2 for 4 days, and Treosulphan 12 gm/M2 for 3 days. GVHD prophylaxis was provided with Methotrexate 10 mg/M2 on days 1, 3 and 6 following transplant, along with Tacrolimus on day 0. Leuconostoc sepsis was treated with intravenous Amoxicillin and Clavulanic acid. Chimerism was assessed using whole blood by fluorescence *in situ* hybridization (FISH) as the transplant was sex mismatched. At 6 months post- transplant, chimerism was down to 28%, and hence 2 donor lymphocyte infusions were given. The last assessment was done at 1 year by T and B cell markers and serum immunoglobulins, and these were found to be within the normal range. The chilld is now one year three months post-transplant and off all medications including immunosuppressive therapy.

The clinical diagnosis of SCID and family history of sibling death prompted us to investigate the molecular genetic correlates of the disease. Since over 13 genes are implicated in SCID and regular molecular testing was not readily available for the genes, we resorted to whole exome sequencing.

## Methods

After obtaining informed consent from the parents, blood was drawn by venipuncture under aseptic precautions. DNA was isolated from whole blood using salting out method ^7^. Exome capture was performed on DNA using the Illumina Nextera rapid capture expanded exome kit using standard protocols (Illumina Inc USA). We generated 47.95 million paired end reads and an average on target coverage of over 25x on Illumina HiSeq 2500 (Illumina Inc. USA). Alignment was performed using BWA (v0.7.12-r1039) ^8^ and Stampy (v1.0.20) ^9^ and variants were called using Platypus (v0.8.1) ^10^. For the prioritisation of variants, we filtered all homozygous variants, further filtered by an allele frequency of <1% in the 1000 Genome and ExAC. Variants in the 13 genes were prioritised and annotated for their deleteriousness using SIFT, Polyphen and Mutation Taster annotations obtained from annovar ^11^.

## Results

Whole exome sequencing analysis revealed a homozygous missense variation (c.2308G>A) in exon number 2 of recombination activating gene 1 ( *RAG1*). The variant was predicted to be highly deleterious by SIFT (score 0.000), PolyPhen2 (0.991) and Mutation Taster (1.00). The variation causes an amino acid change p.E770K, which lies on RAG1 domain of the protein ( Figure 1b). The present variation was not found in the 1000 Genome ( http://browser.1000genomes.org/index.html), ExAC ( http://exac.broadinstitute.org/) or internal control database of over 150 exomes from South East Asian ancestry. Incidentally the mutation was previously reported and analysis suggested a significantly reduced recombination activity ^12^. The variation was further confirmed using targeted PCR amplification around the locus and confirmed by capillary sequencing. The variant was found to be heterozygous in both the parents as well as the surviving siblings ( Figure 1c). The status of the variation could not be ascertained in the sibling who died because no sample was archived and primary immune deficiency was not suspected at the time.

## Discussion

Mutations in recombinase activating gene ( *RAG1)* cause various degrees of severe combined immunodeficiency syndrome (SCID). *RAG1* is involved in the V(D)J recombination ^1, 2, 13^. The child was suspected to have a primary immune deficiency disorder since he had unusually frequent and severe infections and in addition had lost a male sibling due to similar illness. Further, he was born to third degree consanguineous parents. The early onset of symptoms by 2 months of life with increased susceptibility to both bacterial and fungal infections was a pointer to a T cell defect or a phagocytic defect rather than to an antibody deficiency like X linked agammaglobulinemia, which usually presents by 5 to 6 months of age, when maternal antibodies are on the wane ^14^. The immunoglobulin profile showed that there was also a B cell defect. The low absolute lymphocyte counts coupled with radiological evidence of an absent thymus shadow was proof of a T cell defect as well. Thus, a provisional diagnosis of a severe combined immunodeficiency was made even before the flow cytometry results became available and helped confirm the diagnosis.

The possibility of Omenn syndrome was not considered since there was no history of a rash and there was no lymphadenopathy or hepatosplenomegaly, nor was there eosinophilia in the peripheral smear. X-linked recessive severe combined Immunodeficiency (SCID) is characterized by an elevated percentage of B cells and the absence of B cells in the child ruled this out. Janus kinase 3 (Jak3) deficiency was also not thought of for the same reason. Adenosine deaminase (ADA) deficient SCID is characterized by bony abnormalities including rib cage defects, which were absent.

RAG1 or RAG2 deficiencies are associated with a lack of both B cells and T cells and NK cells are predominant in the circulation ^13, 15^. With this possibility in mind, and with a view to offer genetic counselling to the family, whole exome sequencing was considered. Whole exome sequencing identified a mutation c.2308G>A p.E770K in *RAG1*, which was previously reported and shown to significantly reduce recombination activity ^12^. We feel that whole exome sequencing can have more extensive application in the management of primary immune deficiency in developing countries like India, and can add to rapidly expanding scientific knowledge in this arena.

## Ethics approval

The whole exome sequencing was approved by the Institutional Ethical Committee of CSIR - Institute of Genomics and Integrative Biology (IHECC proposal number 8).

## Consent

Written informed consent for publication of the patients’ details and/or their images was obtained from the patients/parents of the patient.

## Data availability

The data referenced by this article are under copyright with the following copyright statement: Copyright: © 2017 Govindaraj GM et al.

All the raw sequencing data are available at the NCBI Sequence Read Archive ( http://www.ncbi.nlm.nih.gov/sra), accession number SRR4088561.
