# Supplementary material for: Case Report: Whole exome sequencing identifies variation c.2308G>A p.E770K in RAG1 associated with B- T- NK+ severe combined immunodeficiency
Source: F1000Res. 2017 Oct 2;5:2532. Originally published 2016 Oct 18. [Version 2] doi: 10.12688/f1000research.9473.2 (PMC5635439; doi:10.12688/f1000research.9473.2)
Supplement: Peer review history

# Peer review: Referee response for version 2

**DOI:** 10.5256/f1000research.13267.r26544

**Published:** 2017-10-10

**Authors:** Paola Itliani

**Affiliations:** 1 Institute of Protein Biochemistry, National Research Council of Italy, Naples, Italy

No further comments.

I have read this submission. I believe that I have an appropriate level of expertise to confirm that it is of an acceptable scientific standard.

# Peer review: Referee response for version 2

**DOI:** 10.5256/f1000research.13267.r26546

**Published:** 2017-10-03

**Authors:** David K. Buchbinder

**Affiliations:** 1 Division of Hematology, Department of Pediatrics, Children's Hospital of Orange County, Orange, CA, USA

Thank you for the edits to the manuscript. I do think this is a very nice case description which highlights the value of next generation approaches in the diagnosis of primary immunodeficiency disorders.  

 My only comments are very minor.  

**Introduction:**

 In the Introduction section I would be review the language and grammar. For example, in the first sentence - "....  of the humoral and/or cellular immune defense mechanism"... sounds awkward. The second sentence may also sound better if you get rid of "the" which starts the sentence. The third sentence where you talk about deficiency of RAG genes being associated with SCID seems awkward. The genes encode the proteins that are deficient. In this case RAG deficiency is associated with T-B-NK+ SCID. I know what you meant, but the sentence is confusing. The next sentence mentions that mutation in RAG1 is associated absence of VDJ recombination. That is not true for all RAG1 variants as you know. Please be careful in your choices of words. The sentence where you talk about an accurate molecular diagnosis being helpful for "genetic counseling for disease" is awkward. Disease what? The last word "gene" (before the sentence "The advent...." should be plural. Lastly, you only need to define the abbreviation RAG1 once.

**Case:**

 You are missing a "he" in the sentence in which you give his birth weight. Define abbreviations like HIV ELISA, AFB, etc. Also, were you able to look at mitogens? That would be useful in defining SCID. What is leuconostoc? What were the doses and timing of the DLI? Any complications such as GVHD? What was the most recent chimerism assessment?  

**Methods:**

 Fine.

**Results:**

 You already defined RAG1.

**Discussion:**

 You already defned RAG1 and SCID. Please use the definition for SCID in the discussion. I would consider "suggested" instead of "was a pointer to". I would get rid of the words "are on the" in front of the word "wane". In the second paragraph you capitalized immunodeficiency. Why? You talk about other forms of SCID such as X-SCID, Jak3, etc. Did you consider other forms of T-B-NK+ SCID? I would consider added this into the discussion.

I have read this submission. I believe that I have an appropriate level of expertise to confirm that it is of an acceptable scientific standard.

# Peer review: Referee response for version 1

**DOI:** 10.5256/f1000research.10202.r20355

**Published:** 2017-02-20

**Authors:** Lennart Hammarström

**Affiliations:** 1 Department of Laboratory Medicine, Karolinska Institutet, Stockholm, Sweden

Govindaraj *et al.* report a typical SCID patient with a known mutation in *Rag1* (p.E770K) with a previously known defective recombination activity and thus, there is very little novel information in this manuscript. In fact, the manuscript is already in itself published in F1000 (October 18th, 2016) and available on the web. The mutation should simply be added to existing databases.

 Additional comments:

- The figure should be improved – providing the different domains.
- The pedigree (inheritance pattern is incomplete) - or simply state that the missing individuals were not sampled.
- Introduction should be more focussed on *Rag*1.
- Immunization schedule (if any) for live vaccines could be of interest for the readers (but not necessary).
- In the methods section, uniformity should apply (cc, mm ul etc). The number of NK cells appear to be too high (higher than the total lymphocyte count).
- Gene names should be in italics and transcript IDs should be given.
- Abbreviations should be correctly used and introduced at the first time of presentation.

I have read this submission. I believe that I have an appropriate level of expertise to state that I do not consider it to be of an acceptable scientific standard, for reasons outlined above.

# Peer review: Referee response for version 1

**DOI:** 10.5256/f1000research.10202.r19588

**Published:** 2017-01-31

**Authors:** David K. Buchbinder

**Affiliations:** 1 Division of Hematology, Department of Pediatrics, Children's Hospital of Orange County, Orange, CA, USA

The authors provide a case report of a patient with severe combined immunodeficiency secondary to RAG deficiency. Whole exome sequencing was utilized to ensure a rapid and accurate diagnosis. Overall, the report is well written. Despite this there are some minor changes that should be considered. I also do not appreciate the novelty of this report and what it adds to the existing literature. The clinical diagnosis of severe combined immunodeficiency is clear. The routine use of next generation sequencing approaches are widely used to provide accurate and rapid diagnoses. 

**Introduction**

- Page 3:  I would consider focusing the introduction on the topic of RAG deficiency.
- Page 3:  I would also note that an accurate and timely diagnosis is vital to the provision of life-saving therapy.
- Page 3:  At the end of the first paragraph the word “sometime” is missing an “s”.
- Page 3:  In the last paragraph of the introduction you talk about a genetic variation E770K. A genetic variation in what gene? Please state *RAG1*.

**Case Report**

- Page 3:  When talking about the patient weight, height, and head circumference I would add in percentiles.
- Page 3:  Please define the abbreviation “PICU”.
- Page 3:  I would review the units on measurements. I would consider using SI units. For example, please review the use of “mg%”. I would address this issue throughout the manuscript.
- Page 3:  How was HIV and tuberculosis excluded?
- Page 3:  Is chest X-Ray correct?
- Page 3:  Was there any evidence of eosinophilia?
- Page 4:  For the immunological evaluation -  were antibody responses assessed? Were RA/RO populations assessed? Were T cell responses to mitogens assessed? Was TCR diversity assessed? Was maternal engraftment assessed?
- Page 4:  Please define the abbreviation “IVIG”.
- Page 4:  How was the CMV infection identified? Where was the “infection”? How was staph pneumonia identified?
- Page 4:  I would consider adding a “Methods” section where you talk about the whole exome techniques used and confirmatory sequencing. I don’t really like how it is right in the middle of the case report.
- Page 4:  In the 4 ^th^ paragraph I would italicize “RAG1”. This should be addressed throughout the manuscript.
- Page 4:  You mention that the mutation was previously reported. This detracts a bit from the novelty. I would also consider noting the RAG activity that was assessed *in-vitro* from the citation.

**Discussion**

- Page 4: You fail to use the abbreviation “SCID” in the first paragraph of the discussion which you defined earlier.
- Page 4: Please define the abbreviations “XR, Jak3, and ADA”.
- Page 4: I would include the portion about the transplant course in the case report section and not at the end of the discussion. I would add in additional details as well -  what type of conditioning was used? Graft-versus-host-disease prophylaxis? What was the etiology of the hypertension? PRES? What was the organism isolated during sepsis? Any other post-transplant issues such as GVHD, VOD, etc.? Was engraftment assessed (i.e. lineage specific chimerism)? 

I have read this submission. I believe that I have an appropriate level of expertise to confirm that it is of an acceptable scientific standard, however I have significant reservations, as outlined above.

# Response

**Published:** 2017-07-26

**Authors:** Vinod Scaria

**Affiliations:** Institute of Genomics and Integrative Biology, Delhi, India

**The authors provide a case report of a patient with severe combined immunodeficiency secondary to RAG deficiency. Whole exome sequencing was utilized to ensure a rapid and accurate diagnosis. Overall, the report is well written. Despite this there are some minor changes that should be considered. I also do not appreciate the novelty of this report and what it adds to the existing literature. The clinical diagnosis of severe combined immunodeficiency is clear. The routine use of next generation sequencing approaches are widely used to provide accurate and rapid diagnoses.**

**Introduction**

- **Page 3: I would consider focusing the introduction on the topic of RAG deficiency.**

Clarification: Suggestions have been incorporated. Introduction is added with focus on RAG1 deficiency.

- **Page 3: I would also note that an accurate and timely diagnosis is vital to the provision of life-saving therapy.**

Clarification: Suggestions have been incorporated.

- **Page 3: At the end of the first paragraph the word “sometime” is missing an “s”.**

Clarification: Corrections has been incorporated.

- **Page 3: In the last paragraph of the introduction you talk about a genetic variation E770K. A genetic variation in what gene? Please state *RAG1*.**

Clarification: Suggestions have been incorporated.

**Case Report**

- **Page 3: When talking about the patient weight, height, and head circumference I would add in percentiles.**

Clarification: His weight, length and head circumference were below the 3 ^rd^ centile as per WHO Child Growth Standards. This is updated in the manuscript.

- **Page 3: Please define the abbreviation “PICU”.**

Clarification: Suggestions have been incorporated.

- **Page 3: I would review the units on measurements. I would consider using SI units. For example, please review the use of “mg%”. I would address this issue throughout the manuscript.**

Clarification: Ionized calcium was 0.28 mmol/L.

- **Page 3: How was HIV and tuberculosis excluded?**

Clarification: The child’s mother was HIV ELISA negative and the child had a negative Mantoux test and negative gastric acid AFB stain.

- **Page 3: Is chest X-Ray correct?**

Clarification: Lateral view of the Chest X Ray.

- **Page 3: Was there any evidence of eosinophilia?**

Clarification: There was no evidence of eosinophilia

- **Page 4: For the immunological evaluation - were antibody responses assessed? Were RA/RO populations assessed? Were T cell responses to mitogens assessed? Was TCR diversity assessed? Was maternal engraftment assessed?**

Clarification: Antibody responses, RA/RO populations, responses to mitogens, TCR diversity and maternal engraftment were not assessed.

- **Page 4: Please define the abbreviation “IVIG”.**

Clarification: Suggestions have been incorporated.

- **Page 4:  How was the CMV infection identified? Where was the “infection”? How was staph pneumonia identified?**

Clarification: CMV infection was identified by DNA PCR. The child had disseminated CMV infection. Staph. pneumonia was identified by characteristic radiological findings and positive blood culture.

- **Page 4: I would consider adding a “Methods” section where you talk about the whole exome techniques used and confirmatory sequencing. I don’t really like how it is right in the middle of the case report.**

Clarification: Suggestions have been incorporated.

- **Page 4: In the 4 ^th ^paragraph I would italicize “RAG1”. This should be addressed throughout the manuscript.**

Clarification: Suggestions have been incorporated.

- **Page 4: You mention that the mutation was previously reported. This detracts a bit from the novelty. I would also consider noting the RAG activity that was assessed *in-vitro *from the citation.**

Clarification: We are not claiming that the mutation is novel. We are pointing that the variation is so rare that it is reported only once. In-vitro assay for RAG activity from citation is noted down in manuscript.

**Discussion**

- **Page 4: You fail to use the abbreviation “SCID” in the first paragraph of the discussion which you defined earlier.**

Clarification: Suggestions have been incorporated.

- **Page 4: Please define the abbreviations “XR, Jak3, and ADA”.**

Clarification: Suggestions have been incorporated.

- **Page 4: I would include the portion about the transplant course in the case report section and not at the end of the discussion. I would add in additional details as well - what type of conditioning was used? Graft-versus-host-disease prophylaxis? What was the etiology of the hypertension? PRES? What was the organism isolated during sepsis? Any other post-transplant issues such as GVHD, VOD, etc.? Was engraftment assessed (i.e. lineage specific chimerism).**

Clarification: Suggestion have been incorporated. The hypertension is an inadvertent typographical error. It should read ‘hypotension’. There was no issue of GVHD or VOD.

# Peer review: Referee response for version 1

**DOI:** 10.5256/f1000research.10202.r17720

**Published:** 2016-11-16

**Authors:** Paola Itliani

**Affiliations:** 1 Institute of Protein Biochemistry, National Research Council of Italy, Naples, Italy

The manuscript “Whole exome sequencing identifies variation c.2308G>A p.E770K in RAG1 associated with B- T- NK+ severe combined immunodeficiency” is the description of a case report, that is a child as a supposed case of SCID. Exactly, the authors found a homozygous variation c.2308G>A by whole exome sequencing and they hypothesized that this variant is associated with the disease. All the sections of the manuscript are well presented and written (title, abstract, content and discussion).

 However, there are two points that I would like to comment:

1. In the introduction the authors wrote that there are over a dozen genes known to be implicated in the disease. So, what about these genes in this case report? By performing a whole exome sequencing, did they also observed the variants of these genes? if yes or not, please comment!
2. In order to explain the functional consequences of the mutated protein, the authors write “The variation causes an amino acid change p.E770K, which lies on RAG1 domain of the protein……Incidentally the mutation was previously reported and analysis suggested a significantly reduced recombination activity” referring to work of Asai E *et al*. Clin Immunol. 2011. In Asai’s work the nucleotide mutation is referred as 2420 G>A with a E770K effect. The position of nucleotide between the two works is different, but the authors of the manuscript declare the same position for amino acid change and the same reduced recombination activity. Please, specify/insert the RS ID number of the homozygous variation.

I have read this submission. I believe that I have an appropriate level of expertise to confirm that it is of an acceptable scientific standard, however I have significant reservations, as outlined above.

# Response

**Published:** 2017-07-26

**Authors:** Vinod Scaria

**Affiliations:** Institute of Genomics and Integrative Biology, Delhi, India

**The manuscript “Whole exome sequencing identifies variation c.2308G>A p.E770K in RAG1 associated with B- T- NK+ severe combined immunodeficiency” is the description of a case report, that is a child as a supposed case of SCID. Exactly, the authors found a homozygous variation c.2308G>A by whole exome sequencing and they hypothesized that this variant is associated with the disease. All the sections of the manuscript are well presented and written (title, abstract, content and discussion).**

**However, there are two points that I would like to comment:**

**1. In the introduction the authors wrote that there are over a dozen genes known to be implicated in the disease. So, what about these genes in this case report? By performing a whole exome sequencing, did they also observed the variants of these genes? if yes or not, please comment**

Clarification 1: We have observed the variations in the genes, which are previously reported with primary immunodeficiency. The prioritized variations are shown in table uploaded in answer to reviewer’s comments. (Sent to editor while uploading revised version). Among the variations, the RAG1:c.2308G>A:p.E770K variation was prioritised due to pathogenic and rarity of variation.

****

**2. In order to explain the functional consequences of the mutated protein, the authors write “The variation causes an amino acid change p.E770K, which lies on RAG1 domain of the protein……Incidentally the mutation was previously reported and analysis suggested a significantly reduced recombination activity” referring to work of Asai E *et al*. Clin Immunol. 2011. In Asai’s work the nucleotide mutation is referred as 2420 G>A with a E770K effect. The position of nucleotide between the two works is different, but the authors of the manuscript declare the same position for amino acid change and the same reduced recombination activity. Please, specify/insert the RS ID number of the homozygous variation.**

Clarification 2: In the original publication by Asai *et al.* 2011, the nucleotide position is mentioned as c.2420G>A. While the position of amino acid is clear from the publication, we are not sure if nucleotide position denotes the position in cDNA or other. In our case, we mentioned the cDNA change as c.2308G>A and amino acid change as p.E770K. We clearly indicated the change in cDNA and it is also the reported nomenclature from database.

For reference, Cosmic mutation ID: COSM3447065.

The rsID for mutation is rs768260595.
